# HOPPSA update: changes in the study protocol of Hysterectomy and OPPortunistic SAlpingectomy, a registry-based randomized controlled trial

**DOI:** 10.1186/s13063-023-07244-w

**Published:** 2023-03-24

**Authors:** Annika Idahl, Per Liv, Anna Darelius, Elin Collins, Karin Sundfeldt, Mathias Pålsson, Annika Strandell

**Affiliations:** 1 Department of Clinical Sciences, Obstetrics and Gynecology, Umeå University, Umeå, Sweden; 2 Department of Public Health and Clinical Medicine, Umeå University, Umeå, Sweden; 3 Department of Obstetrics and Gynecology, Institute of Clinical Sciences, Sahlgrenska Academy at University of Gothenburg, Gothenburg, Sweden

**Keywords:** HOPPSA, Opportunistic salpingectomy, Hysterectomy, Complications, Menopausal symptoms, Ovarian cancer

## Abstract

**Background:**

The HOPPSA trial is a multi-center national registry-based randomized controlled trial to test the safety and effectiveness of performing opportunistic salpingectomy at hysterectomy to reduce the risk of epithelial ovarian cancer (EOC). The study protocol was first published in January 2019 and is available at https://trialsjournal.biomedcentral.com/articles/10.1186/s13063-018-3083-8. Here, we report amendments made to the study protocol since commencement of the trial.

**Changes in methods and analysis:**

The primary outcomes analyses have been changed. (1) *Complications* will be analyzed using binomial generalized estimating equation (GEE) with log link function, while the unadjusted analyses according to Miettinen and Nurminen will be performed as a sensitivity analysis. (2) Absolute change in *Menopause Rating Scale* (MRS) will primarily be analyzed using a mixed effects model, adjusted for baseline MRS and *center* as a random effect. (3) *Time to EOC* will be analyzed using the mixed effects Cox regression model with *center* as random effect, while the unadjusted log-rank test will be performed as a sensitivity analysis.

The primary outcome *Complications* will be based solely on the specific assessment in the GynOp quality registry. The Clavien-Dindo classification will be evaluated as a secondary outcome.

Furthermore, MRS is also measured three years postoperatively to better pinpoint the onset of menopausal symptoms.

**Discussion:**

The changes to the protocol mainly concern the analyses of data. No changes to recruitment, randomization, intervention, or follow-up of primary outcomes have been made. An interim analysis during 2021 concluded that the study should continue until the target sample size is reached.

**Trial registration:**

ClinicalTrials.gov, NCT03045965. Registered 8 February 2017.

## Introduction

Observational studies demonstrate a reduced risk of epithelial ovarian cancer (EOC), both after indicated salpingectomy compared to no surgery and after hysterectomy compared to no surgery [1, 2]. A reduced risk of EOC after opportunistic salpingectomy at the time of hysterectomy compared with hysterectomy alone has yet to be proven. Long-term data on surgical and hormonal safety is insufficient [3]. Therefore, HOPPSA (Hysterectomy and OPPortunistic SAlpingectomy), a Swedish registry-based randomized controlled trial (R-RCT) aiming to analyze the safety and effectiveness of opportunistic salpingectomy at the time of hysterectomy, was started in 2017 (ClinicalTrials.gov, NCT03045965) [4]. Primary outcomes are (1) surgical complications up to 8 weeks postoperatively and (2) menopausal symptoms assessed by the Menopause Rating Scale (MRS) [5] up to 1 year postoperatively, both performed as non-inferiority analyses. Furthermore, (3) EOC incidence up to 30 years postoperatively will be analyzed as a superiority outcome.

During trials that span many years, new evidence or developments in statistical methodology may require a revision of original assumptions. Since the start of the trial in 2017, there has been an ongoing discussion about the statistical analysis approach. These discussions intensified during 2021/2022 and led to the introduction of some changes to the previously described statistical analysis. These changes are presented herein. Furthermore, monitoring of the study provided insights which resulted in small changes to the secondary outcomes, as elaborated below.

## Methods

From 2017 until November 2022, more than 2000 women below the age of 55 were randomized and underwent hysterectomy with or without salpingectomy in the HOPPSA trial in Sweden. Women are identified, randomized, and followed up through the Swedish National Quality Register of Gynecological Surgery (GynOp) [6]. An interim analysis was carried out during 2021, the results of which are known only by the independent data safety monitoring board (DSMB). During the process of managing and monitoring the trial, the following amendments to the initial study protocol have been deemed appropriate. These will improve the value of the final study results.

The study adheres to the EQUATOR network guidelines and the CONSORT statement for R-RCTs.

## Statistical analyses

All primary outcomes will be analyzed using fully conditional multiple imputations to handle missing data. The primary outcomes *complications* and *absolute change in MRS score*, as previously described, will be performed as non-inferiority analysis with the per protocol (PP) approach first and an intention-to-treat (ITT) approach performed as an additional analysis. ITT will be the primary approach in the superiority analysis of the outcome *time to ovarian cancer*. Exploratory prediction model building will be performed as previously described.

1. The outcome *complications* will be evaluated using binomial generalized estimating equation (GEE) with log link function as the primary analysis [7], while the unadjusted analysis according to Miettinen and Nurminen [8] will be performed as a sensitivity analysis. The GEE model will estimate the effect averaged over *center* and will be adjusted for the stratification variables *age* and *surgical route*. The upper (wrongly described as “lower” in the previously published protocol) limit of the 95% CI shall not exceed the non-inferiority margin of 8%. As an additional analysis, complete cases will be analyzed using the GEE method.
2. *Absolute change in MRS score* will be analyzed using a mixed effects model, adjusted for *baseline MRS* and stratification variables [9], with *center* as a random effect. The same method, unadjusted for stratification variables but still adjusted for *baseline MRS*, will be carried out as a sensitivity analysis. The ITT approach, using complete cases and the mixed effects model adjusted for *baseline MRS* and *center* as a random effect, will be carried out as an additional analysis. The upper (wrongly described as “lower” in the previously published protocol) limit of the 95% CI shall not exceed the non-inferiority margin of 4 points.
3. *Time to ovarian cancer* will be analyzed using the mixed effects Cox regression model adjusted for stratification variables with *center* as random effect [9], while the unadjusted log-rank test will be performed as a sensitivity analysis. Complete cases and the PP approach using Cox regression with *center* as random effect will be performed as an additional analysis.

An update to the planned statistical analyses is motivated since the randomization has been stratified by *center*, *age* (< 50/ ≥ 50 years) and *intended operative route* (abdominal, laparoscopic, and vaginal). This is now accounted for in the new planned analyses, using GEE/random effects and covariate adjustments. Not accounting for the stratification variables will render a bias in the standard errors of estimated treatment effects [10]. The full statistical analysis plan (SAP), specifying the planned analyses in detail, will be published before any data are retrieved from the database.

## Other modifications to the study protocol

### Primary outcomes

The primary outcome *complications* will be based on the assessment in the GynOp quality registry according to the specific questions on complications. In the published protocol, the Clavien-Dindo (C-D) classification [11] was included in the primary endpoint *complications*. The C-D classification is only evaluated when a complication is registered in the specific complication question in GynOp. Therefore, this does not add information concerning the primary research question on the difference in number of complications between the treatment arms.

### Secondary outcomes

The Clavien-Dindo classification of surgical complications [11] will be analyzed as a secondary outcome at 8 weeks. The specific complication questions in GynOp include grading of the severity into *no*, *mild*, or *severe* complications. C-D provides an additional perspective of the severity of complications in that it describes the level of intervention needed to treat the complication. In order to use the full description of the complications available, we will include the evaluation of C-D as a secondary outcome.

The questionnaire including MRS and questions regarding hormonal usage, previously described to be administered at 1 and 5 years postoperatively, will also be administered to the participants at 3 years postoperatively to better pinpoint the onset of menopausal symptoms.

Complications at 1 year: *Persistent symptoms related to surgery* and *patient satisfaction with surgery* will be analyzed as secondary outcomes at 1 year. These outcomes are routinely collected in the GynOp registry and have been of value for assessing the quality of gynecological surgical routines in Sweden.

## Discussion

The HOPPSA trial is still actively recruiting participants to elucidate the surgical and hormonal safety of opportunistic salpingectomy at hysterectomy and the long-term effectiveness at reducing the risk of EOC. The changes to the protocol presented herein have evolved over time and without any knowledge of the accumulating results or outcome. Changes concern the statistical analyses to better account for stratification variables and covariate adjustments. No changes were made to the recruitment process, randomization, intervention, follow-up, or sample size. In November 2020, half of the estimated target sample size for the outcome *complications* was recruited and an interim analysis was performed in March 2021 by an independent data safety monitoring board (DSMB). The DSMB recommended that the HOPPSA steering group should continue recruiting until the target sample size is reached, estimated by the beginning of 2024 for both complications and MRS evaluation.

Despite the lack of robust evidence for safety and effect size, opportunistic salpingectomy has increasingly been implemented at hysterectomy as a preventive procedure for EOC. Recent observational data shows conflicting results regarding the effects on ovarian function. Women in one study had an increased risk for climacteric symptoms 1 year after opportunistic salpingectomy [12]. In another study with a 5-year follow-up period, a shorter time from surgery to menopause after opportunistic salpingectomy was found [13]. On the contrary, time to physician visit for menopausal symptoms or time to prescription of menopausal hormone therapy was not shorter in women having opportunistic salpingectomy in another large observational study, however with shorter follow-up [14]. A systematic review concluded that in the short term, using biochemical indicators or antral follicle count, there is no significant reduction in ovarian reserve after opportunistic salpingectomy, but it may cause earlier onset of menopause [15]. However, clear evidence of the long-term effects on age at onset of menopause are lacking [15]. Surgical complications were not increased, but the certainty of evidence was graded as *very low* or *low* in a systematic review [16]. Observational data with follow-up after 2 weeks reported an increased use of analgesics but not physician visits for surgical infections, complications, laboratory tests, or requests for imaging [17].

No randomized trial has reported the ovarian cancer incidence after opportunistic salpingectomy [3]. Promising results regarding risk of ovarian cancer were recently presented in a retrospective observational cohort study from British Columbia, Canada [18]. Even though the study is well-designed, drawbacks include short follow-up in a younger population and an observational design which is prone to residual confounding.

HOPPSA will be the first large population-based randomized controlled trial with long-term follow-up evaluating clinically relevant patient reported safety outcomes, as well as the effectiveness of opportunistic salpingectomy at hysterectomy. No other ongoing randomized clinical trials on this topic have been found in ClinicalTrials. A general search in other databases for clinical research protocols gave no results. Currently, 45 out of 48 Swedish gynecological clinics, with a coverage of 90% of hysterectomies on benign indication in Sweden, have recruited participants and contributed to the study. The results will lead to important evidence which will inform women about the short-term safety and risks of having salpingectomy at hysterectomy. This increases the potential for shared decision making. If there are no increased risks, we can recommend all women to have opportunistic salpingectomy. If there are, the risks and the uncertainty of the benefits will be shared and, together with patient preferences, this enables personalized care.

In conclusion, changes to the initial study protocol of HOPPSA, a registry-based randomized controlled trial of opportunistic salpingectomy at hysterectomy, are presented herein. No changes to recruitment, randomization, intervention or follow-up of primary outcomes have been made. The amendments concern data analyses to better reflect the current state of the art for RCT analysis.

## Data Availability

Not applicable.

## Abbreviations

C-D: Clavien-Dindo
DSMB: Data safety monitoring board
EOC: Epithelial ovarian cancer
GEE: Generalized estimating equation
GynOp: The Swedish National Quality Register of Gynecological Surgery
HOPPSA: Hysterectomy and OPPortunistic SAlpingectomy
ITT: Intention to treat
MRS: Menopause Rating Scale
PP: Per protocol
R-RCT: Registry-based randomized controlled trial
SAP: Statistical analysis plan
